# Analysis of Aftershocks from California and Synthetic Series by Using Visibility Graph Algorithm

**DOI:** 10.3390/e27020178

**Published:** 2025-02-08

**Authors:** Alejandro Muñoz-Diosdado, Ana María Aguilar-Molina, Eric Eduardo Solis-Montufar, José Alberto Zamora-Justo

**Affiliations:** Unidad Profesional Interdisciplinaria de Biotecnología, Instituto Politécnico Nacional, Mexico City 07340, Mexico; amunozdi@ipn.mx (A.M.-D.); anafrom@hotmail.com (A.M.A.-M.); eric.montufar@gmail.com (E.E.S.-M.)

**Keywords:** seismicity series, seismicity from California, spring-block model, visibility graph algorithm, complex networks

## Abstract

The use of the Visibility Graph Algorithm (VGA) has proven to be a valuable tool for analyzing both real and synthetic seismicity series. Specifically, VGA transforms time series into a network representation in which structural properties such as node connectivity, clustering, and community structure can be quantitatively measured, thereby revealing underlying correlations and dynamics that may remain hidden in traditional linear or spectral analyses. The time series transformation into complex networks with VGA provides a new approach to analyze seismic dynamics, allowing scientists to extract trends and behaviors that may not be possible by classical time-series analysis. On the other hand, many studies attempt to find viable trends in order to identify preparation mechanisms prior to a strong earthquake or to analyze the aftershocks. In this work, the seismic activity of Southern California Earthquake was analyzed focusing only on the significant earthquakes. For this purpose, seismic series preceding and following each earthquake were constructed using a windowing method with different overlaps and the slope of the connectivity (*k*) versus magnitude (*M*) graph (*k-M* slope) and the average degree were computed from the mapped complex networks. The results revealed a significant decrease in these parameters after the earthquake, due to the contribution of the aftershocks from the main event. Interestingly, the study was extended to synthetic seismicity series and the same behavior was observed for both *k-M* slope and average degree. This finding suggests that the spring-block model reproduces a relaxation mechanism following a large-magnitude event like those of real seismic aftershocks. However, this conclusion contrasts with conclusions drawn by other researchers. These results highlight the utility of VGA in studying events that precede and follow major earthquakes. This technique may be used to extract some useful trends in seismicity, which could eventually be employed for a deeper understanding and possible forecasting of seismic behavior.

## 1. Introduction

Complex systems comprise numerous elements that interact non-linearly, exchange information with the environment, and continuously modify their internal structure and activity patterns through self-organization, a distinguishing property of these systems. As a result, they are flexible and easily adapt to changing external conditions [1].

Self-organized criticality (*SOC*) is a concept related to conservation laws in complex systems. It was introduced by Bak, Tang, and Wiesenfeld to describe the appearance of scale invariance in nature. Earthquakes are an example of a self-organized critical (SOC) phenomenon in nature and exhibit power law behavior [2,3]. Power laws describe the behavior of earthquakes and the distribution of their magnitude. The movement of continental plates is caused by convection currents in the Earth’s mantle. These currents generate displacements and collisions between the plates, which causes the accumulation of tension at their limits. When the accumulated tension exceeds the resistance of the rocks, it is released abruptly, causing an earthquake. In this process, the release of energy causes a rebound or sudden displacement of the plates, which generates seismic waves that propagate through the Earth. Earthquakes are the result of the dynamic organization of the Earth’s crust and the fault system that forms it. The large-scale structure of the crust is the product of a historical accumulation of seismic events that have modified and weakened rocks over time [3,4].

Researchers studying earthquakes seek specific mechanisms underlying large events, frequently describing each occurrence individually and independently of others. Seismic events adhere to a simple distribution known as the Gutenberg-Richter law. This law illustrates the frequency of earthquakes at each magnitude in a region, representing the energy released during seismic events. The Gutenberg-Richter law indicates that large or small earthquakes have the same rules. Therefore, this law serves as evidence that the Earth’s crust has self-organized until it reaches a critical state [1,5,6,7].

Olami, Feder, and Christensen (*OFC*) have studied self-organized criticality (*SOC*) in non-conservative systems using an earthquake model. They showed that a two-dimensional spring-block model for earthquakes is equivalent to a non-conservative continuum cellular automaton model. The model exhibits power-law distributions for the energy released during an earthquake and characteristics similar of real earthquakes: the occurrence of small earthquakes is random, while larger earthquakes appear to be clustered. The simplest test for these models is their ability to reproduce the Gutenberg-Richter law [8,9].

Network analysis, based on concepts from statistical physics and graph theory, can be useful in characterizing the irregular behavior of seismicity time series [10,11]. This approach consists of transforming a discrete set of data into a network consisting of nodes and the links between them. Converting a time series into a network facilitates the exploration of complex system dynamics through analysis of network properties [12,13]. Several methods have been proposed to convert time series into networks and to characterize these series in terms of the parameters that describe the network. For instance, Zhang and Small [14] transformed periodic time series into networks by representing each cycle as a node. Connections between nodes were established based on the similarity or difference between cycles. Marwan et al. [15] constructed univariate recurrence networks by determining whether points in a phase space reconstruction of the time series are proximate to each other. If they are, a link is created between the corresponding nodes. Furthermore, Gao et al. [16] proposed an extension of univariate recurrence networks to multiple time series, linking nodes based on proximity in a high-dimensional phase space.

A key method in this area is the visibility graph algorithm (VGA) [17], which transforms a time series into a complex network while preserving its fundamental characteristics, thereby enabling the application of network theory to characterize the original data. This mapping transforms the dynamic characteristics of the time series into features of the resultant network. In this method, each data point in the time series is treated as a node. Two nodes are connected by an edge if they can “see” each other, meaning there exists a direct line of sight between them that does not intersect any intermediate data points. In VGA, the degree of connectivity represents the number of connections per node. This algorithm has recently been applied to various fields, including heart rate variability analysis [18], medical science [19,20,21,22], and particularly real and synthetic seismicity series [12,13,23,24,25,26,27,28].

For instance, Muñoz-Diosdado et al. [18] identified significant differences in the *k-M* parameter, average degree, and average path length of complex networks derived from heart rate variability series of subjects under various exercise and health conditions.

In seismicity research, Telesca et al. [23] applied the VGA method to analyze sequences of seismic magnitudes, revealing a power-law distribution in the connectivity degrees. This scale-free pattern in connectivity aligns with other scale-invariant features observed in earthquake studies. Earthquakes demonstrate complex spatial and temporal behavior, with numerous seismological laws exhibiting fractal characteristics, as indicated by scale exponents [29,30].

In the visibility graph methodology, the degree of connectivity, *k*, is defined for each event based on its magnitude *M* and its position in the time series. The *k-M* parameter represents the slope of the linear regression of the graph plotting *k* against *M*. Telesca et al. [12] performed a similar analysis on regional seismicity in Pannonia, proposing that the correlation between these two parameters is nearly universal. Analogous results were obtained when analyzing experimentally derived seismicity time series [12]. Further studies in this direction were conducted by Azizzadeh-Roodpish et al. [27,31] for Southern California and the Alaskan Crustal and Aleutian subduction zones, by Khoshnevis et al. [26] for the seismicity of northern Iran, and by Pérez-Oregon et al. [28] using synthetic earthquake time series. These studies collectively reinforced the notion of a universal linear correlation.

In this study, we analyzed the complex network parameters *k-M* slope and average degree using VGA by using real seismicity data from California and synthetic seismicity generated with the spring-block model. The series were selected based on high-magnitude earthquakes, and windowing was applied to include all events before and after each main earthquake. This approach allowed us to observe how these parameters change within the dynamics of seismicity, particularly during aftershock sequences.

## 2. Materials and Methods

### 2.1. Visibility Graph Algorithm

**Figure 1:**
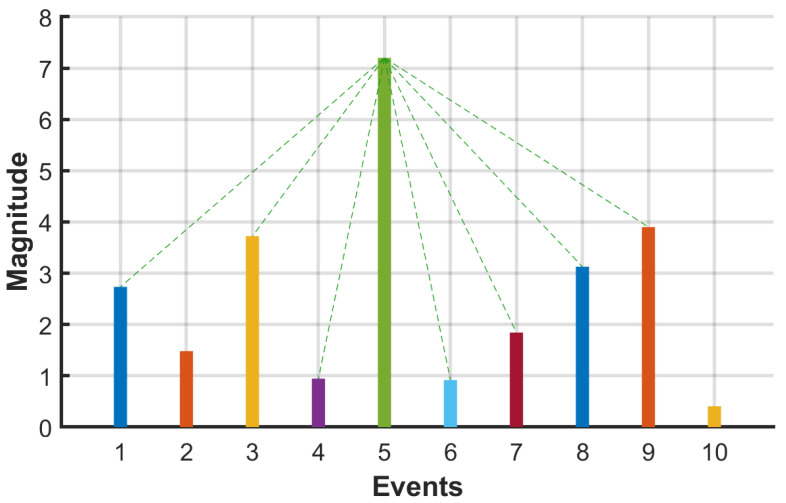
Explanation of the visibility graph algorithm. Each event is represented by a node in the visibility graph. Two nodes are connected if the straight line joining them is not intersected by another event. In this figure, it can be seen the connected events (green dashed line).

The visibility graph algorithm (VGA) was used to transform the seismicity time series into a complex network. In the undirected visibility graph (VG), two events are connected if they can “see” each other, meaning that no event between them intersects the line connecting them (see Figure 1). By mapping the time series into a graph with interconnected nodes, the resulting complex network retains various properties of the time series [17]. Let x(i),1≤i≤N be a seismicity time series of length *N*, and consider each earthquake as a node in the VG. Nodes *x(i)* and *x(j)* (i≠j) are connected only when the following condition is met [17]:

(1) $$\forall l\in \left[i,j\right];x\left(l\right)<x\left(i\right)-\left(j-l\right)\frac{x(j)-x(i)}{j-1}.$$

Adjacency matrices were constructed for the visibility graph of the seismicity time series. The dimension of the adjacency matrix corresponds to the number of nodes in the network. If there is a connection between the nodes represented by a specific row and column, the matrix element is one; otherwise, it is zero. For an undirected visibility graph, this matrix is symmetric, with the main diagonal elements being zero. Typically, there are ones around the main diagonal since neighboring events are more likely to be visible to each other. The degree of connectivity for each node is determined by summing the number of connections in the corresponding row of the matrix [17].

In seismicity analysis, the *k-M* refers to the slope of the linear regression of the graph plotting the connectivity degree *k* against the earthquake magnitude *M*. It is generally observed that periods of lower seismic activity correspond to a low connectivity degree *k*, while periods of higher seismic activity are associated with a higher connectivity degree. Consequently, the *k-M* slope typically has a positive value [32].

### 2.2. California Seismicity

The first systematic, non-mythical treatment of earthquakes comes from Greece. With the spread of writing, people began to collect descriptions of severe earthquakes. In California, this documentation began around 1800 by the Franciscan Fathers, who documented the development of Spanish missions. By the 19th century, the documentation was quite detailed. Seismographic stations were installed in many parts of the world at the beginning of the 20th century. These sensitive seismographs record small seismic waves [33].

The importance of a global network of seismographs lies in documenting earthquakes worldwide so that they are no longer limited to subjective reports of observed effects. From waves recorded at different seismographic observatories, it is possible to calculate the position of the epicenter of an earthquake, which provides a uniform representation of the distribution of earthquakes.

The seismotectonics of Southern California are shaped by the region’s complex tectonic setting, which is dominated by the Pacific and North American plate boundary. This interaction creates a highly active and intricate fault network, making Southern California one of the most seismically active regions in the world. The area is particularly well known for the San Andreas Fault, a transform fault that accommodates much of the relative plate motion and poses a significant seismic hazard [34].

**Figure 2:**
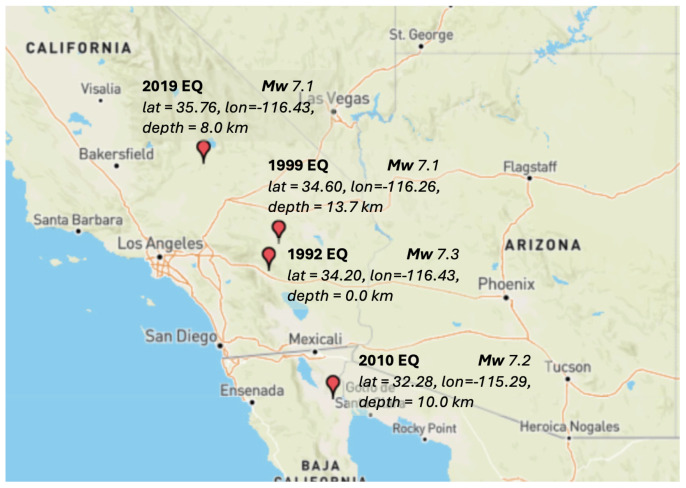
The map showing the locations of earthquakes with a magnitude of 7 or greater from the southern California catalog.

In the Figure 2, it can be observed epicenters of the earthquakes in Southern California with magnitude ≥ 7 between 1980 and 2024.

The southern section of the San Andreas Fault (*SSAF*) is the only historically active segment of the SSAF system, which is believed to represent the greatest seismic risk in California. Concerns about the timing, magnitude, and location of future earthquakes, coupled with the fault’s accessibility, have yielded evidence of prehistoric earthquakes [35,36,37].

It was analyzed the earthquake catalog of Southern California for the period from 1980 to 2024, which was obtained from the website “The Southern California Earthquake Data Center” (*SCEDC*), which operates at the Seismological Laboratory at Caltech and is the primary archive of seismic data for Southern California. The downloaded catalog contains a total of 671,533 events. However, the catalog is complete for magnitudes starting at 1.5, with a total of 284,197 earthquakes remaining in this range. It is important to note that no depth discrimination was performed in this analysis.

From the Catalog, it was identified earthquakes with a magnitude of 7.0 or greater, finding four such events. The first occurred 10 km north of Yucca Valley, California, on 28 June 1992, with a magnitude of 7.3. The second occurred 16 km southwest of Ludlow, California, on 16 October 1999, with a magnitude of 7.1. The third occurred 12 km southwest of Delta, Baja California, Mexico, on 4 April 2010, with a magnitude of 7.2. Finally, the fourth occurred 18 km west of Searles Valley, California, on 6 July 2019, with a magnitude of 7.1 (see Table 1 and Figure 2 for detailed information on each earthquake). After locating the earthquakes with magnitudes of 7 or greater, we selected 50 windows before and after each event with $2^{10}$ data. It is worth mentioning that exploration circles of 100 km radius around the main earthquakes were used, helping to exclude distant events.

**Figure 3:**
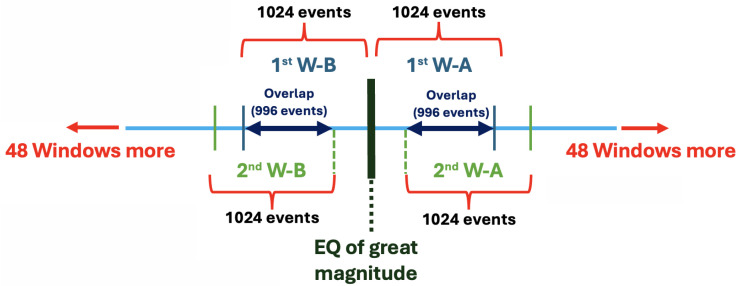
Illustration of the windowing process applied to the seismicity series. The series was divided into overlapping windows of 1024 events before and after the earthquake of great magnitude, with labels 1st W-B and 1st W-A for the first windows before and after the event, and 2nd W-B and 2nd W-A for the second windows before and after.

Figure 3 represents how the windowing process was applied to the seismicity series. The light blue line shows a fragment of this series, where the earthquake (EQ) of great magnitude is located in the middle to perform the windowing. This procedure consists of dividing the series into consecutive windows of 1024 events selected before and after the earthquake. In the figure, the first window before the earthquake is labeled as 1st W-B (First Window-Before), while the first window after the earthquake is labeled as 1st W-A (First Window-After). The double arrow line represents the overlap of 996 events between consecutive windows. To ensure that each window contains exactly 1024 events, the data is completed using adjacent events in the series, i.e., events immediately before or after the current window, if necessary. Subsequent windows are named following the same scheme. For example, the second window before the earthquake is labeled as 2nd W-B (Second Window-Before), and the second window after the earthquake is labeled 2nd W-A (Second Window-After). This procedure is systematically repeated to generate a total of 50 windows before and 50 windows after the selected earthquake. The goal of this method is to analyze the variations in the characteristics of the seismicity series around the main event. This allows for the identification of patterns or anomalies, such as possible seismic precursors, changes in event frequency, or unusual behaviors that could be relevant for the study.

It is worth mentioning that in this study initially the overlap of the windows was varied, however, the one that yielded the best results was with 996 data.

**Figure 4:**
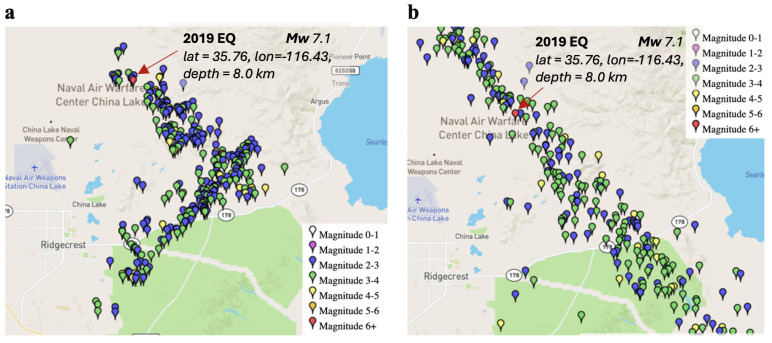
Illustration of the spatial variation of the events included (**a**) before and (**b**) after the 2019 earthquake. Only events with a magnitude of 2.5 or greater are shown.

Figure 4 illustrates some events occurred before and after the 2019 earthquake, which was considered in the aforementioned windowing process. It can be observed that prior to this earthquake, normal seismic activity in Southern California is observed; however, after the earthquake, aftershocks are evident along the San Andreas Fault.

Finally, the obtained series of each window were analyzed by using VGA and the *k-M* slope and average degree were calculated.

### 2.3. Spring-Block Method

Earthquakes obey the Gutenberg-Richter law, which is the distribution of energy released during an earthquake [5,38]. In 1956 they discovered the relationship [5,38]:

(2) $$\log_{10}N\left(M>m\right)=a-bm.$$

where *M* is magnitude, *N*(*M*) is the number of earthquakes of magnitude ≦ *M*, *a* is a constant related to the overall seismic activity in the region and determines the frequency of earthquakes in that area, and *b* determines the relative number of earthquakes of different magnitudes. The Equation (2) is the Gutenberg-Richter law. The parameter *a* and *b* depend on the location, but *b* has been observed to vary significantly across different faults. Values of *b* have been reported ranging from 0.80 to 1.06 for great magnitude earthquakes and from 1.23 to 1.54 for small magnitude earthquakes [5,7,38].

The energy *E* released during the earthquake increases exponentially with the size of the earthquake as follows [39]:

(3) $$\log_{10}E=c+dm$$

where *d* is 1 and 3/2 for small and large earthquakes, respectively [8,38,39,40]. So, the Gutenberg-Richter law is essentially a power law connecting the frequency distribution function with the energy release *E*

(4) $$N\left(E_{0}>E\right)∼E^{-b/d}=E^{-B}$$

*B* is in the same range for both small and large earthquakes, namely, 0.80–1.05 [39]. the power law that exhibits the Gutenberg-Richter is related to geometric features of the fault structure and Mandelbrot noted the earthquakes occur on “fractal” self-similar sets [5,38].

The Olami, Feder, and Christensen (OFC) spring-block model [38] is a two-dimensional system of blocks interconnected by springs. Each block is connected to its four nearest neighbors and to a single rigid drive plate by another set of springs, as well as frictionally connected to a fixed rigid plate. The blocks are driven by the relative motion of the two rigid plates. When the force on one of the blocks is greater than the maximum static friction, the block slides.

The OFC model can be mapped to a cellular automaton model that uses an L×L matrix of blocks identified by their coordinates (i,j), where *i*,*j* are integers within the interval [1,L]. The displacement of each block from its relaxed position in the grid is denoted as $dx_{i,j}$. The total force exerted by the springs on a given block (i,j) is expressed as:

(5) $$F_{i,j}=K_{1}\left[2dx_{i,j}-dx_{i-1,j}-dx_{i+1,j}\right]+K_{2}\left[2dx_{i,j}-dx_{i,j-1}-dx_{i,j+1}\right]+K_{L}dx_{i,j}$$

where $K_{1}$, $K_{2}$ and $K_{L}$ represent the spring constants.

When the two rigid plates move relative to each other, the total force on each block increases uniformly at a rate proportional to $K_{L}V$, where *V* is the relative velocity between the two plates. This increase continues until a site reaches a threshold value, at which point the relaxation process begins, triggering a synthetic earthquake.

The redistribution of stress after a local slip at position (i,j) is described by the following relations:

(6) $$\begin{array}{c} F_{i\pm 1,j}\to F_{i\pm 1,j}+\delta F_{i\pm 1,j},\\ F_{i,j\pm 1}\to F_{i,j\pm 1}+\delta F_{i,j\pm 1},\\ F_{i,j}\to 0 \end{array}$$

where the increments in the forces of the nearest-neighbor blocks are given by:

(7) $$\begin{array}{c} \delta F_{i\pm 1,j}=\frac{K_{1}}{2K_{1}+2K_{2}+K_{L}}F_{i,j}=\beta_{1}F_{i,j}\\ \delta F_{i\pm 1,j}=\frac{K_{1}}{2K_{1}+2K_{2}+K_{L}}F_{i,j}=\beta_{1}F_{i,j} \end{array}$$

For simplicity, the elastic redistribution coefficients are denoted as $\beta_{1}$ and $\beta_{2}$, respectively, although the isotropic case is generally considered, that is, $\beta_{1}=\beta_{2}=\beta$. If we assume that all elastic constants are on the same scale ($K_{1}\approx K_{2}\approx K_{L}$) then β=0.2.

The total number of slips following a single initial slip event is a measure of the size (seismic moment) of the earthquake. *OFC* measure the probability distribution of the size (the total number of relaxations) of the earthquakes, which is proportional to the energy released during an earthquake. We used the OFC model to generate a time series of 10,000,000 data with a conservation level of β=0.2 for a 100×100 blocks. The series contains data on the number of earthquakes and the number of relaxed blocks. To model the relationship between magnitude and event frequency, the following expression was used:

(8) $$log_{3}N=M$$

where *N* is the number of events and *M* is the magnitude.

This relationship allows for the adjustment of the magnitude distribution in the synthetic series. Earthquakes with magnitudes greater than 7.0 were excluded from the analysis because synthetic seismicity produces a large number of events of that magnitude, making it challenging to identify specific patterns due to their high frequency. We focused on earthquakes with magnitudes greater than 8.0, which represent the largest and least frequent events. In this case, it was found only three earthquakes of that magnitude, allowing for a more detailed analysis of their characteristics. It is worth mentioning that these identified synthetic earthquakes are well-separated from each other, ensuring no overlap between them. Similarly, for California seismicity, we analyzed the most significant recorded earthquakes, enabling comparisons between the behavior of extreme events in both synthetic and real scenarios, identifying similarities and differences in the distribution and dynamics of these events.

Therefore, we were interested in studying the dynamics that occur before and after an earthquake, as well as the behavior of aftershocks in real seismicity and synthetic seismicity. Then, once the earthquakes with a magnitude of 8.0 or greater were located, we constructed 50 windows before and after each earthquake with $2^{10}$ data by using an overlap of 996 events (see Figure 3). Additionally, in the case of synthetic catalogs, since they are complete, a threshold magnitude is not considered (lines 100), unlike the California catalog where it was set at 1.5. Finally, we applied VGA to these time series and calculate the *k-M* and average degree.

## 3. Results

### 3.1. Seismicity from California

**Figure 5:**
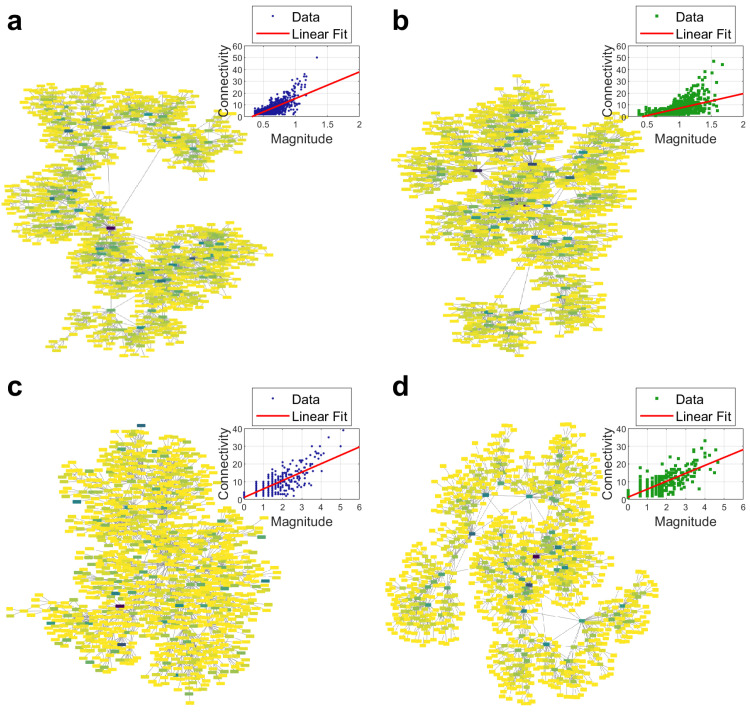
Illustration of the complex network and connectivity vs. magnitude graph formed by the seismicity series from California (**a**) before and (**b**) after a great earthquake, as well as the complex network and *k-M* plots formed by synthetic seismicity series (**c**) before and (**d**) after the earthquake. It can be observed that before the earthquake, the networks form fewer clusters of larger size, while those networks after the earthquake form a greater number of clusters with fewer nodes.

The California seismicity series were analyzed using VGA, where the earthquakes were mapped to nodes in a visibility graph. In this approach, each node’s connectivity *k* is obtained by counting how many other nodes (earthquakes) it is directly visible to in the adjacency matrix of the visibility graph. Specifically, each row of the adjacency matrix reflects a node’s links to all other nodes, so the degree *k* is the sum of those links. Figure 5 shows a typical representation of the complex network formed by series before and after the earthquake, respectively. In the visibility graphs of Figure 5a (before the earthquake), we see a higher concentration of highly connected nodes, forming a more compact structure with larger clusters. By contrast, in the Figure 5a (after the earthquake), the average degree clearly decreases: there are more dispersed clusters with fewer nodes in each cluster, and fewer nodes exhibiting very high connectivity. In terms of visibility graphs, this reduction can be explained by the clustering of aftershocks following the main event. Aftershocks tend to occur in rapid succession, which affects the “lines of sight” between seismic events in the time series—many smaller events in a short period often create shorter visibility spans, leading to a noticeable shift in the node degree distribution toward lower connectivity. Thus, the underlying seismic dynamics, dominated by aftershocks, become reflected in a sparser, more fragmented visibility graph structure.

**Figure 6:**
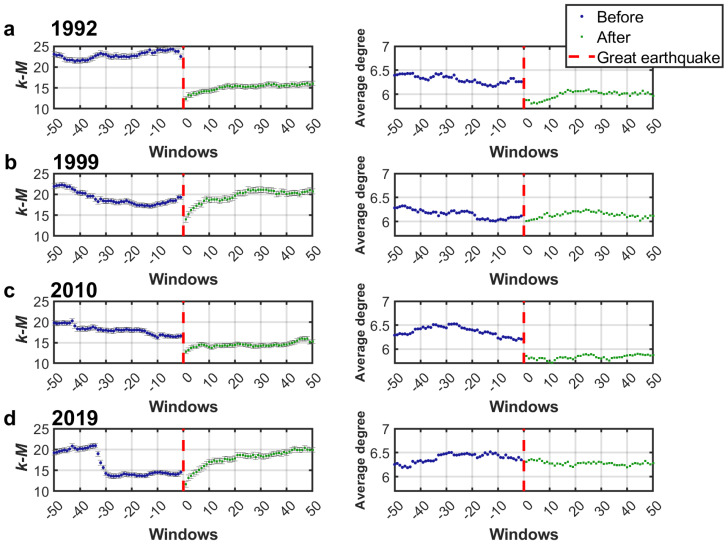
*k-M* slope and average degree values obtained from California seismicity series of the (**a**) 1992, (**b**) 1999, (**c**) 2010, and (**d**) 2019 earthquakes.

First, the slope of the graph of connectivity vs magnitude at each node (*k-M* slope) was analyzed. Figure 6 shows the results for the 50 windows before and after the earthquake for the four earthquakes studied. It can be observed that before the earthquake the *k-M* values are around 20, however in the following window after the great earthquake these values decrease.

It is noteworthy that for the 2019 earthquake the *k-M* actually increases considerably after the great earthquake (Figure 6d), this is attributed to the fact that in window 32 before this great earthquake an event of magnitude 6.4 had already occurred nearby (information about this event: date 2019/07/04, $M_{w}=6.4$, lat=35.70533, lon=−117.50383, and depth=10.5 km). Such mid-sequence events can transiently alter connectivity and raise or lower *k-M* values as aftershocks subside and the network reconfigures. Furthermore, the aftershocks of a magnitude 6.4 earthquake are smaller in magnitude and fewer in number compared to those generated by a magnitude 7.1 earthquake, so the value of *k-M* will decrease further once a larger magnitude earthquake occurs. This fact is further strengthened by the graph where it can be observed how the *k-M* values decrease in this window and decrease even more after the earthquake of magnitude 7.1.

Furthermore, Figure 6 shows the error bars corresponding to the calculation of the *k-M* slope from the linear fit by least squares. It can be observed that the errors represent around 2.5% of the calculated *k-M* values, this confirms that the errors in the slope calculation are not significant, which further strengthens the findings described above.

This study was now repeated considering the calculation of the average degree which can be observed in the Figure 6. Here the same trend was observed, that is, the values decrease significantly after the earthquake. It should be noted that the average degree of the second earthquake increases after the earthquake instead of decreasing.

Overall, the observed post-earthquake decrease in *k-M* slope and in average degree suggests a transient disruption in connectivity patterns, likely influenced by the clustering of aftershocks. Over time, these values may recover or even exceed pre-earthquake levels, reflecting how the visibility network reconfigures as seismic activity stabilizes.

### 3.2. Seismicity from Synthetic Series

Once it was confirmed that in real seismicity there is a significant change in the parameters of complex networks studied for real seismicity, we were interested in verifying whether this trend can also be reproduced by synthetic series of seismicity, so we repeated the same analysis with the series obtained with the OFC model. Especially because we noticed that the representation of the complex networks shown in the insets c and d of Figure 5 also exhibit differences between the series before and after the earthquake. In that figure, it can be observed that before the earthquake the network forms a cluster with many nodes, whereas after the earthquake the network forms smaller clusters.

**Figure 7:**
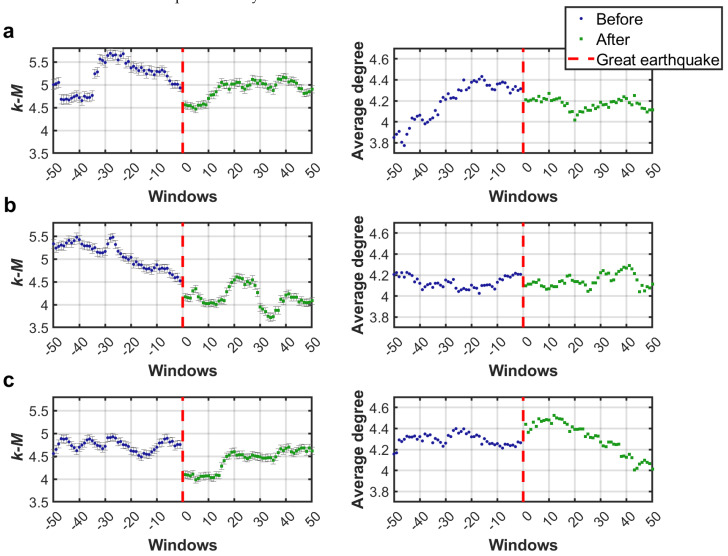
*k-M* slope and average degree values obtained from synthetic seismicity series of the (**a**) earthquake 1, (**b**) earthquake 2, and (**c**) earthquake 3.

Figure 7 shows the values of *k-M* and average degree obtained for three synthetic series of seismicity. Although these values were not in the same range than those obtained for the California seismic series, with this analysis it can again be observed that both parameters decrease after the main earthquake. Newly, the error bars for the calculation of *k-M* values are shown in Figure 7. It can be observed that these errors are not significant before or after the earthquake, which suggests that the same behavior observed in real seismicity was reproduced by the OFC model.

## 4. Discussion

The results suggest that the methods implemented in this work show changes in the dynamics before and after the great earthquake, as reported in [41]. Specially, it was observed that there is a significant change in the *k-M* slope in the windows before and after the three California’s earthquakes analyzed. Specifically, the network parameter *k-M* decreases, which is due to a reduction in node connectivity caused by the presence of aftershocks. These aftershocks are more frequent but lower in magnitude, leading to a loss of network connectivity. Additionally, we found that this change is also preserved when analyzing synthetic seismicity using the spring-block model, which suggests that such relaxation mechanisms can also be reproduced by this model, contrary to what has been indicated in other studies [41].

It should be remembered that, in the spring-block model, and specifically in its non-conservative variant Olami−Feder−Christensen (OFC), the aftershock process is not explicitly modeled. However, we observe variations in the results that suggest the possible existence of aftershocks due to the partial redistribution of stresses. The model consists of placing two parallel plates: one mobile and one fixed, to which small blocks are attached by means of springs. When moving the mobile plate, one of the blocks exceeds its threshold force, triggering the continuous movement of the neighboring blocks. During this process, the energy is redistributed towards the adjacent blocks, leaving some of them “loaded” with residual energy. This is manifested in the series of synthetic earthquakes as aftershocks, observed in the graphs and in the multifractal analysis carried out in [38,41].

It is well known that the calculation of the *k-M* slope can be imprecise due to the wide dispersion of points in the connectivity vs. magnitude plot. As a result, after fitting, the correlation coefficient may not be significant. However, to corroborate the aforementioned findings, we also decided to calculate the average degree, which is somewhat related to *k-M* as it helps to measure the average connectivity level of all nodes [17,42]. This analysis revealed that the decrease in the *k-M* slope after the earthquake persists, reaffirming this trend caused by the aftershocks.

Before a large earthquake, *k-M* values correspond to low seismic activity. However, when a large earthquake occurs, the number of aftershocks, which are events of higher magnitude, causes a decrease in connectivity and therefore in *k-M*. Subsequently, after the aftershocks, normal seismic activity is reestablished, causing *k-M* values to rise again. Interestingly, the same pattern was observed in the analysis of synthetic series, once again suggesting that these relaxation mechanisms are also present in the spring-block model.

It is worth mentioning that the initial proposal included the analysis of additional complex network parameters, such as the average path length. However, we found that the differences in the values of these parameters were not significant. This may be because the networks formed from the windowed data exhibit small-world characteristics, which are typically defined by relatively short average path lengths coexisting with high clustering coefficients [43]. In such networks, even when nodes are rearranged or new connections appear, the overall path length may remain consistently short, making it challenging to observe subtle changes in dynamics between events. Consequently, the impact of an earthquake on certain small-world-like properties may be overshadowed by the inherent structural robustness of these networks. Nevertheless, future studies could explore a broader range of parameters to identify new trends that might help detect aftershocks or even preparation mechanisms before a great earthquake occurs.

In addition, the analysis presented highlights a significant similarity in the behavior of the parameters derived from complex networks for both real and synthetic seismicity, particularly in the context of large-magnitude events and their aftershocks. The process of overlapping windowing before and after these earthquakes allows for a detailed evaluation of the evolution of the clustering coefficient *k-M* and the average degree of the networks generated using the visibility algorithm.

The observation of an increase in the *k-M* value in real seismicity, as a consequence of the rise in the number of earthquakes due to aftershocks, is consistent with the physics of the process: the triggering of a secondary seismic sequence enhances the connectivity of the associated networks, reflecting an increase in local interactions between events. The fact that this same behavior is observed in synthetic seismicity generated through the OFC model suggests that this model adequately captures the essential dynamics of stress generation and relaxation in seismic systems.

The finding of aftershocks in synthetic large-magnitude earthquakes within the OFC model can be interpreted as evidence that the relaxation of blocks following a main event generates near-rupture conditions in some blocks within the affected area. This critical state promotes the occurrence of new events, analogous to aftershocks in natural seismicity. These results strengthen the relevance of the OFC model for studying not only main events but also post-seismic dynamics, paving the way for a more detailed analysis of stress conditions and their evolution after a great earthquake.

## 5. Conclusions

The largest earthquakes were identified in both synthetic seismicity and real seismicity in Southern California. Taking each of these large-magnitude earthquakes as a reference, a window superposition process was applied before and after the main event. Each window represents a subsequence to which the visibility algorithm was applied and two parameters of the resulting complex networks were calculated, *k-M* and the average degree. This allowed us to observe the evolution of these parameters before and after the earthquake.

There is a difference in the behavior of these parameters in the seismicity of Southern California before and after four earthquakes that were the largest between 1992 and 2019, which is due to the presence of their aftershocks. It is important to note that the same trend is observed in synthetic seismicity, which leads us to propose that there is also aftershock-like behavior in the *OFC* model after large synthetic earthquakes. This is because after a large synthetic earthquake, a significant number of blocks relax. These blocks represent the “rupture zone” of the synthetic earthquake, and it appears that many of these blocks remain in a state of stress very close to the threshold. It is therefore reasonable to expect high activity in the blocks within the synthetic earthquake rupture area after a major event, which is related to aftershocks in real seismicity.

## Figures and Tables

**Table 1:** The information on earthquakes with a magnitude of 7 or greater from the California catalog for the period from 1 January 1980 to 19 August 2019.

YYY/MM/DD	HH:mm:SS.ss	Magnitude $\left(M_{w}\right)$	Latitude	Longitude	Depth (km)
1992/06/28	11:57:34.13	7.3	34.20000	−116.43700	0.0
1999/10/16	09:46:44.46	7.1	34.60333	−116.26500	13.7
2010/04/04	22:40:42.36	7.2	32.28617	−115.29533	10.0
2019/07/06	03:19:53.04	7.1	35.76950	−117.59933	8.0

## Data Availability

All data used in the present work could be requested to correspondence author.

## Abbreviations

The following abbreviations are used in this manuscript:

EQ	Earthquake
OFC	Olami−Feder−Christensen’s model
SCEDF	The Southern California Earthquake Data Center
SSAF	Southern San Andreas Fault
VG	Visibility Graph
VGA	Visibility Graph Algorithm
